# Identification and Validation of Immune Implication of R-Spondin 1 and an R-Spondin 1-Related Prognostic Signature in Esophagus Cancer

**DOI:** 10.1155/2024/7974277

**Published:** 2024-05-29

**Authors:** Yuansheng Lin, Xinqi Lou, Shengjun Li, Wei Cai, Tuanjie Che

**Affiliations:** ^1^ Department of Intensive Care Unit Suzhou Hospital Affiliated Hospital of Medical School Nanjing University, Suzhou 215000, China; ^2^ Institute of Clinical Medicine Research Suzhou Hospital Affiliated Hospital of Medical School Nanjing University, Suzhou 215000, China; ^3^ Department of Emergency and Critical Care Medicine Suzhou Hospital Affiliated Hospital of Medical School Nanjing University, Suzhou 215000, China; ^4^ The Open Project of Key Laboratory of Functional Genomics and Molecular Diagnosis of Gansu Province, Lanzhou 730000, China

## Abstract

R-spondin 1 (RSPO1), which encodes a secretory-activating protein, is a promising therapeutic target for various tumors. The aim of this study was to establish a robust RSPO1-related signature specific to esophageal cancer (ESCA). Our comprehensive study involved meticulous analysis of RSPO1 expression in ESCA tissues and validation across ESCA cell lines and clinical samples using The Cancer Genome Atlas (TCGA) and GTEx databases. Using TCGA-ESCA dataset, we employed single-sample gene set enrichment analysis (ssGSEA) to elucidate the complex interaction between RSPO1 expression and the abundance of 22 specific immune cell types infiltrating ESCA. The biological significance of RSPO1 was further elucidated using KEGG, GO, and GSEA, demonstrating its relevance to pivotal tumor and immune pathways. This study culminated in the construction of prognostic nomograms enriched by calibration curves, facilitating the projection of individual survival probabilities at intervals of one, three, and five years. A substantial decrease in RSPO1 expression was observed within ESCA tissues and cell lines compared to their normal esophageal counterparts, and a significant decrease in the proportion of activated dendritic cells was evident within ESCA, accompanied by an augmented presence of macrophages and naive B cells relative to normal tissue. GSEA and KEGG analyses showed that RSPO1 was associated with tumor and immune pathways. Additionally, an independent prognostic risk score based on the RSPO1-related gene signature was developed and validated for patients with ESCA. Finally, RT-qPCR and western blotting were performed to confirm RSPO1 expression in normal and ESCA cell lines and tissue samples. In summary, our investigation underscores the pivotal role of RSPO1 in orchestrating tumor immunity and proposes RSPO1 as a prospective target for immunotherapeutic interventions in ESCA. Furthermore, the intricate profile of the two RSPO1-related genes has emerged as a promising predictive biomarker with notable potential for application in ESCA.

## 1. Introduction

Esophageal cancer (ESCA) is one of the most prevalent malignancies and a substantial hazard to human health worldwide. The most recent global cancer incidence and mortality statistics rank ESCA ninth in incidence and sixth in mortality [1]. In China, ESCA is the third and fourth leading cause of malignant tumor mortality and incidence, respectively [2]. Despite advances in ESCA diagnosis and treatment, the overall survival (OS) rate remains poor due to the high recurrence rate following treatment. Therefore, there is a need to identify effective biomarkers and potential molecular targets to improve therapeutic strategies for patients with ESCA.

Chemotherapy is the main treatment method for ESCA, and resistance to chemotherapeutic drugs is an important obstacle to effective treatment. However, HMGA1 can drive chemotherapy resistance in ESCA by inhibiting ferroptosis [3]. Extracellular vesicles (EVs) are small extracellular vesicles. EVs derived from cancer cells promote tumor progression and metastasis. To improve outcomes in patients with cancer, researchers have proposed various strategies for targeting cancer-derived EVs, including inhibiting the production of EVs, destroying EVs during transport, and blocking the uptake of EVs by recipient cells [4]. In the treatment of gastric and gastroesophageal junction adenocarcinoma, with the new adjuvant atezolizumab combined with chemotherapy, atezolizumab can lead to immune activation of the tumor microenvironment (TME), which promotes better patient prognosis [5]. However, the mechanism underlying the role of R-spondin 1 (RSPO1) in tumor immunology remains unclear.

RSPO1, a member of the RSPO family, has a molecular weight of 35 kDa and is associated with the activation of Wnt signaling [6, 7]. Lgr5 homolog is a component of the facultative Wnt receptor, and Wnt signal enhancement is mediated by soluble R-spondin protein. The WNT/*β*-catenin signal is associated with the development of tumor [8]. Numerous studies have demonstrated that RSPO1 contributes to cancer progression. RSPO1 plays a role in the progression of cancers, such as colorectal [9], breast [10], liver [11], glioma [12], and ovarian [13]. Moreover, the immune-related mechanisms of RSPO1 and its prognostic signature have not yet been studied.

In this study, we investigated the RSPO1 expression and the association between RSPO1 expression and immunity in patients with ESCA. RSPO1 may be a viable immunotherapeutic target for ESCA. Finally, we generated prognostic immune signals using RSPO1-related genes and constructed nomograms by integrating two RSPO1-related gene signatures and clinical features.

## 2. Material and Methods

### 2.1. RNA-Sequencing Data

The Cancer Genome Atlas (TCGA) (https://portal.gdc.com) was used to retrieve the RNA-seq data and clinical information of 173 patients (11 normal and 162 patients with ESCA) [14]. Gene expression data of the cell lines were acquired using the Cancer Cell Line Encyclopedia (CCLE) [15]. Tissues from two patients were collected for experimental verification.

### 2.2. Genomic Characteristics of RSPO1

The chromosomal localization information for RSPO1 was obtained from the GeneCards database. The protein structure of RSPO1 was obtained from the Human Protein Atlas (HPA) database.

### 2.3. Mutation and Methylation Characteristics of RSPO1

cBioPortal was used to analyze the mutation frequency of RSPO1 in ESCA. Differences in RSPO1 methylation between ESCA and healthy tissue samples were analyzed using the UALCAN database. Additionally, R was used to analyze the levels of the 12 methylation sites of RSPO1. Finally, we explored the prognostic value of the RSPO1 methylation sites.

### 2.4. Identification of Tumor-Infiltrating Immune Cells in TCGA-ESCA

We employed the CIBERSORT technique to detect 22 distinct types of immunocytes in tissues, including T cells, plasma cells, NK cells, naïve and memory B cells, and myeloid subsets [16]. Matrix mRNA levels were assessed using the CIBERSORT R script (https://cibersort.stanford.edu/), with the CIBERSORT L22 dataset as a comparative reference.

### 2.5. Correlation between RSPO1 and Immune Cell Infiltration

The ESTIMATE R package was used to predict stromal and immunocyte infiltration into the tumor tissues. This involved estimating stromal and immunocyte content in malignant tumor tissue using gene expression data, yielding three scores: (I) ImmuneScore, reflecting immune cell infiltration; (II) StromalScore, indicating stroma presence; and (III) ESTIMATEScore, inferring tumor purity. Pearson's correlation coefficient was used to examine the association between RSPO1 and immune cells. The Tumor Immune Estimation Resource (TIMER) provides comprehensive tumor immunocyte analysis (http://cistrome.dfci.harvard.edu/TIMER/) [17].

### 2.6. Cell Culture

The Chinese Academy of Sciences Cell Bank supplied human ESCA TE12 and EC109 cells and esophageal epithelial HEEC cells (Fuheng Biology, Shanghai, China). TE12 cells and HEEC were cultured in high-glucose DMEM supplemented with 10% fetal bovine serum and 1% penicillin/streptomycin. EC109 cells were cultured in RPMI-1640 supplemented with 10% fetal bovine serum and 1% penicillin/streptomycin. At 37°C and 5% CO_2_, the cells were incubated in an incubator.

### 2.7. RT-qPCR

Total RNA was extracted from the tissues following the manufacturer's guidelines using TRIzol reagent (Invitrogen, USA). The cDNA was reverse transcribed using M-MLV reverse transcriptase (Promega). RT-qPCR was performed on a StepOnePlus real-time PCR system (ABI, USA) using a SYBR Premix Ex Taq kit (TaKaRa, Japan).

The primer sequences are as follows: RSPO1 forward primer, 5′-GAGGTGTTAGCAAGAGCCGTGTG-3′, and RSPO1 reverse primer, 5′-CAGCCCAAGCCGCATAGTCAC-3′, and *β*-actin forward primer, 5′-GCACCACACCTTCTACAATGAGC-3′, and *β*-actin reverse primer, 5′-GGATAGCACAGCCTGGATAGCAAC-3′. The relative expression of RSPO1 was calculated by the 2^-ΔΔCt^ comparison method.

### 2.8. Western Blot Analysis

RIPA lysis buffer (Biosharp Company, Shanghai, China) was used for separation and assessment. Equal amounts of protein were isolated using a rapid dispensing kit (10%) and then deposited onto polyvinylidene fluoride membranes. RSPO1 antibodies were identified by primary antibodies: rabbit anti-human *β*-actin antibody (Abways, China, AB0035, RRID:AB_2828227) (42 kDa; 1 : 1000), mouse anti-human *α*-Tubulin (Immunoway, YM3035, RRID:AB_10598496) (52 kDa; 1 : 1000), mouse anti-human RSPO1 monoclonal antibody (Immunoway, USA, YM0566, RRID:AB_10978732) (28.9 kDa; 1 : 1000) at 4°C overnight following a 1 h blocking step at room temperature using 5% skim milk.

### 2.9. The Biological Significance of RSPO1 Expression in Tumors

We extracted and analyzed ESCA cell line RNA-seq data from the CCLE database. The RSPO1 expression profile was retrieved for further analysis. Pathways showed a correlation with RSPO1 expression (RSPO1 high vs. RSPO1 low) by using GSEA 4.0.2 software [18].

### 2.10. Survival Analysis

We aimed to build a prognostic gene signature based on RSPO1-coexpressed genes. Cox model variables were selected in a stepwise manner using the Akaike information criterion [19]. Following the selection of coexpressed genes, the prognosis index is referred to as the risk score: risk score = *β*1*X*1 + *β*2*X*2 + ⋯+*βiXi*, where *Xi* is the expression level of each gene and *βi* is the Cox model-derived risk coefficient for each gene. Using the Kaplan-Meier survival curve, log-rank test, and univariate Cox analysis, we evaluated the correlation between the RSPO1 coexpression gene signature and clinical parameters, as well as OS. Risk scores for TNM stage, T, N, M, age, and sex were adjusted using multivariate analysis. The receiver operating characteristic (ROC) curves were utilized using the survivalROC software to determine the prognostic accuracy of the risk score [20].

### 2.11. Nomogram Development

We generated a prognostic nomogram by integrating the patients' clinical attributes and risk assessments. The concordance index was used to assess the predictive accuracy of the nomograms.

### 2.12. Statistics

All statistical analyses were conducted using R 4.2.1. For survival analysis, the K-M curve expression along with the log-rank test was employed, while Spearman's correlation analysis was used for survival assessment. Forest plot, rms, pheatmap, timeROC, GSEABase, and ggplot2 are related R packages. Statistical significance was set at *p* < 0.05, indicating statistical significance.

## 3. Results

### 3.1. Genomic Characteristics of RSPO1

**Figure 1:**
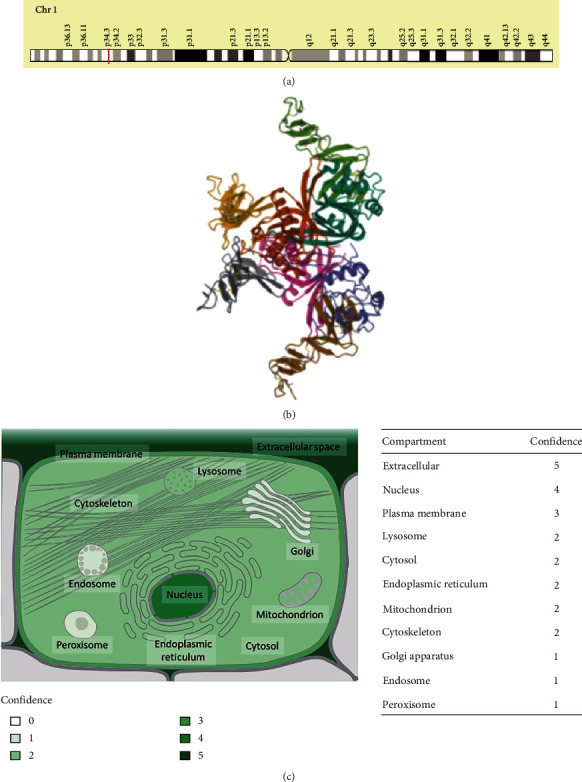
Genomic characteristics of RSPO1. (a) Chromosome localization of RSPO1 in human. (b) The protein structure of RSPO1. (c) The location of RSPO1 protein in cells.

The human *RSPO1*gene is located in the p34.3 region of chromosome 1 and has a length of 23,543 bp (Figure 1(a)). The three-dimensional structure of the RSPO1 protein is predicted through the NCBI database (Figure 1(b)). The HPA database indicated that the subcellular localization of RSPO1 was mainly in the extracellular space (Figure 1(c)).

### 3.2. Identification of *RSPO1* Expression in ESCA Tissues and Cell Lines

**Figure 2:**
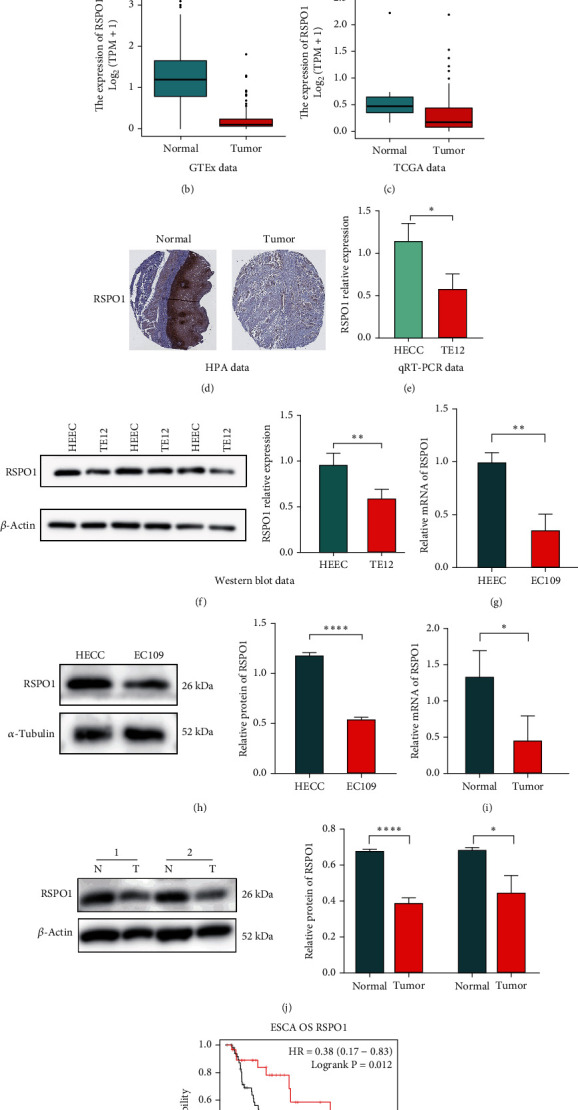
Differences in expression of RSPO1. (a) RSPO1 expression levels in tumor tissues detected via the TIMER database. (b) Expression of RSPO1 in GTEx data. (c) Expression of RSPO1 in TCGA data. (d) Immunohistochemical staining for RSPO1 in normal esophagus tissues and ESCA tissues from the HPA. (e) The mRNA level of RSPO1 was downregulated in TE12 cell of ESCA by RT-qPCR experiment. (f) Left: the protein level of RSPO1 was downregulated in TE12 cell of ESCA by WB experiment. Right: the statistical graph of WB. (g) Left: WB analysis of RSPO1 level in HEEC and EC109 cells. Right: the statistical graph of WB. (h) RT-qPCR was used to detect the expression level of RSPO1 mRNA in HEEC and EC109 cells. (i) Left: the protein level of RSPO1 in tissue by WB. Right: the statistical graph of WB. (j) RT-qPCR was used to detect the RSPO1 mRNA expression in tissue. (k) Survival analysis of ESCA patients with low and high RSPO1 expression in the Kaplan-Meier plotter database. ^∗∗∗∗^*p* < 0.0001, ^∗∗∗^*p* < 0.001, ^∗∗^*p* < 0.01, and ^∗^*p* < 0.05.

In TIMER database analysis, 14 types of tumor tissues (including ESCA tissues) showed decreased RSPO1 mRNA levels compared with those of normal tissues (Figure 2(a)). The decreased expression of RSPO1 mRNA in ESCA tissues was further confirmed using the GTEx and TCGA databases (Figures 2(b) and 2(c)). Moreover, immunohistochemical data from the HPA demonstrated higher levels of RSPO1 expression in normal esophageal tissues than in ESCA tissues (Figure 2(d)). To confirm these observations, qRT-PCR and western blotting were conducted on ESCA cells, which consistently revealed decreased RSPO1 mRNA levels compared to those in normal cells (Figures 2(e) and 2(f)). Notably, similar trends were observed in the qRT-PCR and western blot analyses of EC109 tissues (Figures 2(g), 2(h), 2(i), and 2(j)). Furthermore, OS analysis of patients with ESCA revealed that those with high RSPO1 expression had significantly better survival rates than those with low RSPO1 expression (Figure 2(k)).

### 3.3. Mutation and Methylation Characteristics of RSPO1

**Figure 3:**
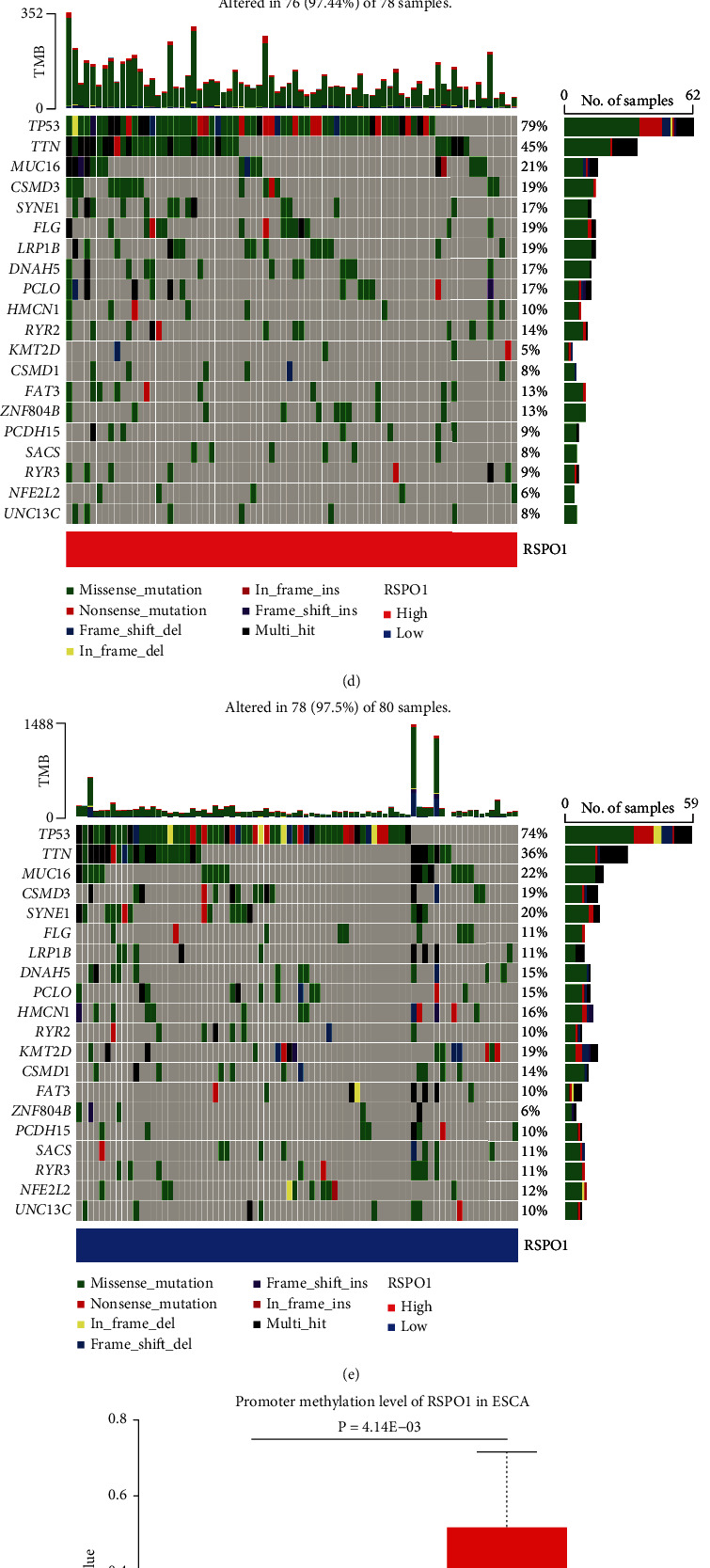
Mutation and methylation of RSPO1 based on TCGA-ESCA. (a) Alterations in RSPO1 expression in ESCA. (b) The alteration frequency with mutation type. (c) The sites of RSPO1 genetic mutations in amino acid sequence. (d, e) Detection of differential somatic mutations in ESCA between RSPO1 high group and RSPO1 low group. (f) Methylation level of RSPO1 in healthy and ESCA. (g) Correlation analysis between RSPO1 expression and the cg26519919 site. (h) Survival analysis of ESCA patients with cg22063989 high and cg22063989 low in TCGA-ESCA dataset.

Genetic alterations are associated with the development of malignant tumors, and mutated genes may become targets for cancer therapy. Given that RSPO1 mutations may be molecular targets for cancer treatment, we analyzed ESCA samples from TCGA. We found that 1.5% of the samples had RSPO1 mutations, and the main changes in RSPO1 were amplification and missense mutations (Figure 3(a)). The mutation frequency of RSPO1 was highest (>3%) in TCGA gastric ESCA samples, with the majority being of the “amplification” type. All samples of ESCA were of the “amplification” type (Figure 3(b)). The types and sites of RSPO1 mutations were distributed throughout the amino acid sequence, with the V72E site being the most frequently detected in ESCA (Figure 3(c)). We also studied the association between RSPO1 expression and specific genomic features by focusing on somatic mutations and copy number variations in the TCGA-ESCA dataset. In this analysis, the high RSPO1 expression group (*n* = 78) showed more frequent somatic mutations in genes, such as *TP53* (79%) and *TTN* (41%). In contrast, the low RSPO1 expression group (*n* = 78) showed a higher incidence of somatic mutations in *TP53* (74%), *TTN* (36%), and *MUC16* (22%) (Figures 3(d) and 3(e)). These results indicate that the different mutation characteristics of RSPO1 in ESCA may be associated with the abnormal expression of RSPO1.

To explore whether DNA methylation of *RSPO1* was associated with tumorigenesis, we used TCGA database to assess the methylation level of the RSPO1 promoter. The results suggested that the methylation status of *RSPO1* was higher in ESCA tissues than in the corresponding normal tissues (Figure 3(f)). Subsequently, we explored the relation between the methylation site of RSPO1 and its expression. The results suggested that cg26519919 positively correlated with RSPO1 expression (Figure 3(g)). Finally, we analyzed the prognostic significance of cg26519919 in ESCA. It was found that high expression of the cg26519919 site was associated with a better prognosis (Figure 3(h)).

### 3.4. Correlation between RSPO1 Expression and Immune Infiltration

**Figure 4:**
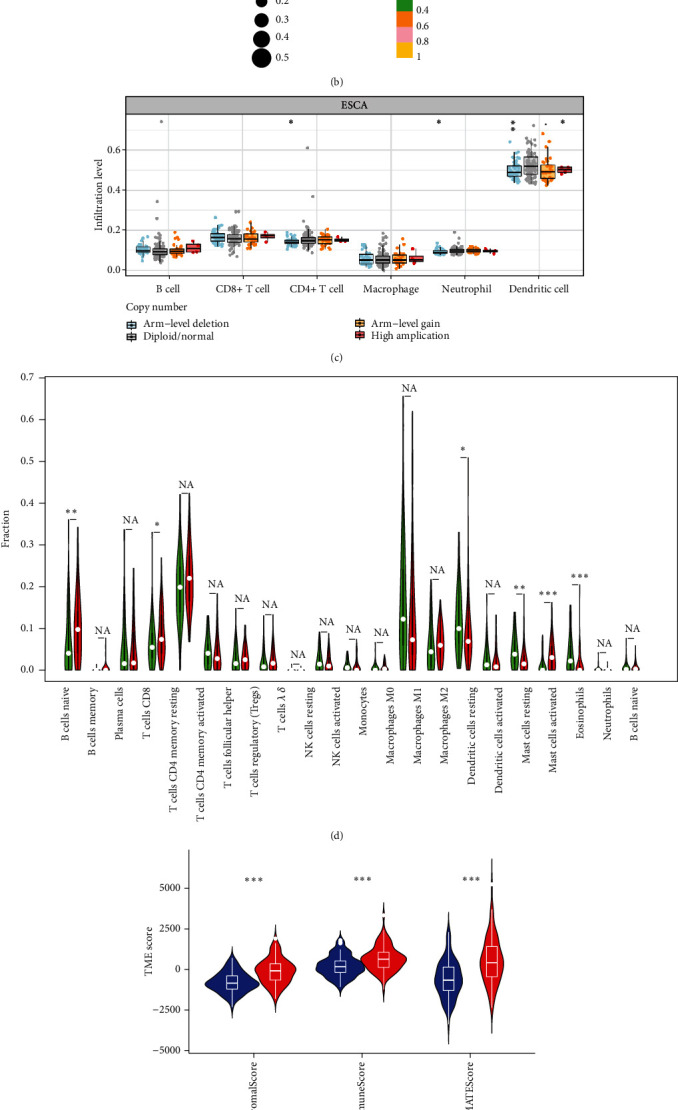
Correlation between RSPO1 expression and immune infiltration. (a) The expression of RSPO1 in ESCA tissues is related to immune cell infiltration. (b) Relationship between RSPO1 expression and 22 tumor-infiltrating lymphocytes. (c) RSPO1 copy number variation affects the infiltration level of immune cells in ESCA. (d) The proportion of 22 immune cell types in RSPO1 high expression group and low expression group was compared. (e) The immune infiltration level of high RSPO1 expression group showed higher StromalScore, ImmuneScore, and ESTIMATEScore than the low expression group. (f) Heatmap of the correlation between RSPO1 and tumor immune checkpoint molecules. *p* value significant codes: ^∗∗∗^*p* < 0.001, ^∗∗^*p* < 0.01, and ^∗^*p* < 0.05.

Given the significance of immune infiltration in ESCA development [21], TIMER2.0 was used to evaluate the relation between RSPO1 expression and ESCA immune cell infiltration. The results showed that RSPO1 was associated with the degree of B cell, CD4+ T cell, and macrophage infiltration (Figure 4(a)). Using ssGSEA, further analyses were performed to explore the relation between RSPO1 expression and the infiltration levels of 22 distinct immune cell types in ESCA. RSPO1 expression in ESCA positively correlated with the infiltration of resting mast cells, CD8+ T cells, naive B cells, macrophages M1, T cells CD4 memory resting, and monocytes. Conversely, RSPO1 expression negatively correlated with the abundance of activated NK cells, macrophages M0, mast cells activated, and activated dendritic cells (Figure 4(b)). Notably, changes in immune cell infiltration levels aligned with RSPO1 copy number alterations. Infiltration levels of some immune cells, such as those of CD4+ T cells, neutrophils, and dendritic cells, are associated with RSPO1 copy number variability in ESCA. This suggests that immune cell infiltration is associated with copy number variations of RSPO1 (Figure 4(c)). To further investigate the interaction between RSPO1 expression and the TME, we used the CIBERSORT algorithm to focus on the tumor-infiltrating immune cells of ESCA samples. First, by comparing the proportions of 22 immune cell types between the RSPO1 high and low expression groups, six tumor-infiltrating immune cells showed significant differences (Figure 4(d)). Furthermore, we investigated the association between RSPO1 expression and the immunological environment (including stromal score, immune score, and estimated score). The TME score can be used to predict the effects of immune checkpoint therapy in ESCA. A high score corresponds to a response to treatment and has good predictive accuracy [22]. The TME scores, including the stromal score, immune score, and estimate score, were elevated in the high RSPO1 expression group compared with those in the low RSPO1 expression group. These results emphasized the immunotherapeutic potential of elevated RSPO1expression, indicating its correlation with improved TME conditions (Figure 4(e)). To identify a more precise immunotherapeutic target for ESCA, we investigated the link between RSPO1 and tumor-infiltrating lymphocyte surface markers (Figure 4(f)). Together, these findings suggest that RSPO1 plays important roles in tumor immunity.

### 3.5. The Prognostic Implication of RSPO1-Coexpressed Genes in ESCA

**Figure 5:**
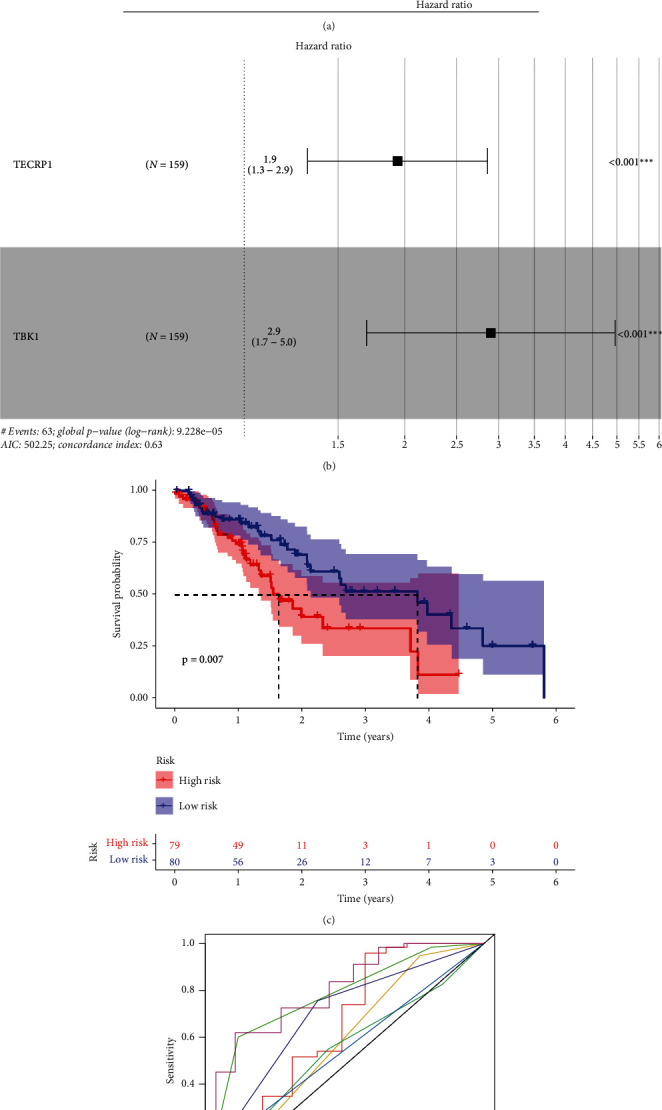
The construction of prognostic signatures based on RSPO1-coexpressed genes and RSPO1. (a, b) Univariate and multivariate Cox regression analyses were used to evaluate the relationship between these genes and OS in the forest plots for ESCA. (c) Kaplan-Meier curves for ESCA regarding the risk scores. (d) AUC values of the risk, gender, and stage.

**Figure 6:**
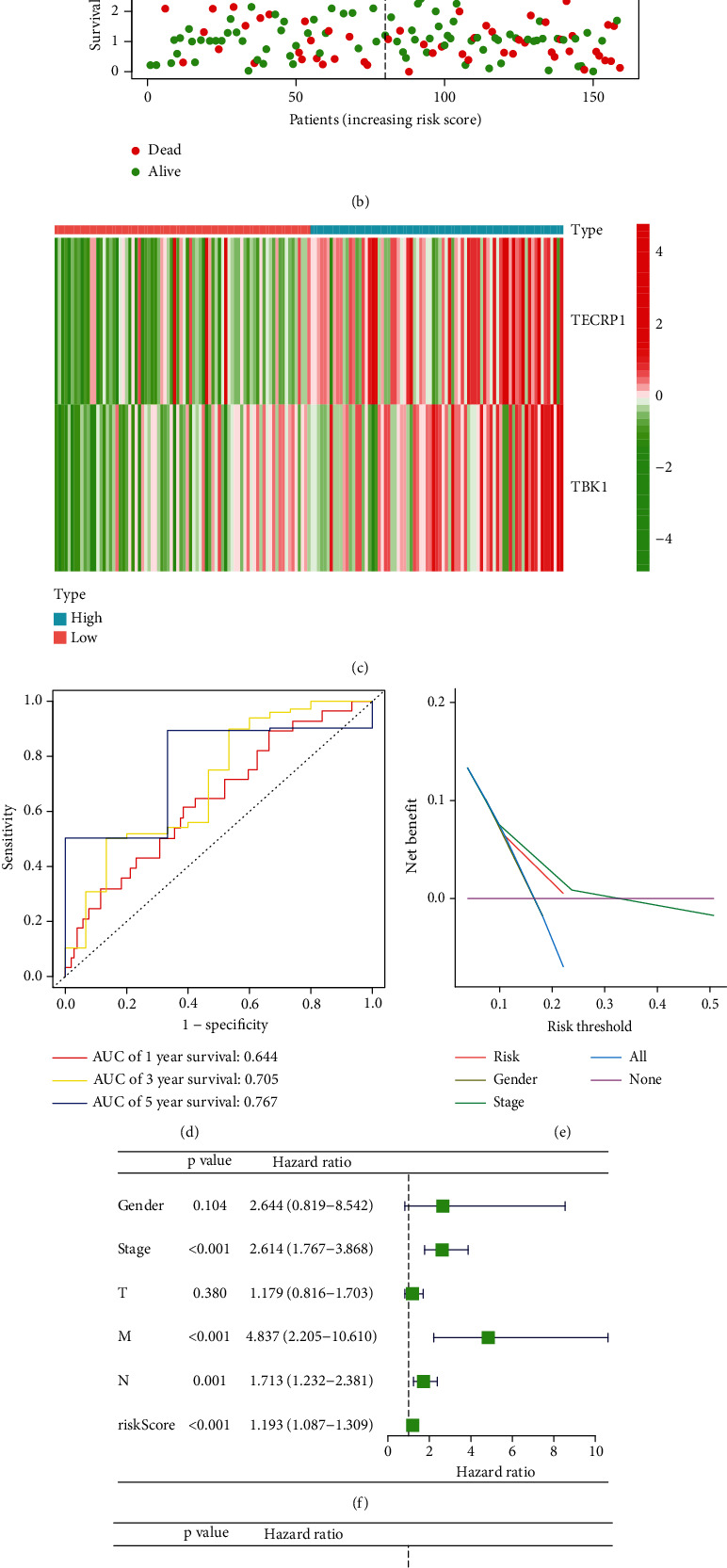
Prognostic values of the risk scores in TCGA-ESCA cohorts. (a–c) Distribution of risk scores, survival statuses, and gene expression profiles for ESCA. (d, e) ROC curve and DCA analysis to evaluate the predictive performance of the constructed nomogram. (f, g) Univariate and multivariate Cox regression analyses of the risk score in ESCA.

Furthermore, we explored the relation between the 44 genes coexpressed with RSPO1 and the CCLE data in ESCA. We analyzed the association between these genes and OS using univariate and multivariate Cox regression analyses. Two optimal prognostic signature genes for ESCA were identified using this method (Figures 5(a) and 5(b)). Spearman's method was used to evaluate the correlation among RSPO1, TECRP1, and TBK1. Spearman's correlation analysis showed that RSPO1 was significantly correlated with TECRP1 and TBK1 (Supplementary Figure 1). Patients with high risk scores had shorter survival times than those with low risk scores (log-rank test, *p* = 0.007) (Figure 5(c)). The areas under the curve for risk, sex, and stage were 0.660, 0.593, and 0.798, respectively. When risk and clinical data were merged, an area under the curve of 0.82 was found (Figure 5(d)). The frequency distribution of ESCA risk score, survival status, and hallmark gene expression profiles is shown (Figures 6(a), 6(b), and 6(c)). Furthermore, we employed ROC curve and decision curve analysis to thoroughly assess the predictive performance of the formulated nomogram. The ROC curve and decision curve analyses both underscored the robust predictive probability of the constructed RSPO1-related prognostic signature (Figures 6(d) and 6(e)). In TCGA-ESCA univariate and multivariate Cox regression models, the risk score was significantly linked to survival (hazard ratio = 1.193, 95%confidence interval = 1.087–1.309, *p* < 0.001 and hazard ratio = 1.211, 95%confidence interval = 1.107–1.325, *p* < 0.001, respectively) (Figures 6(f) and 6(g)). These results indicated that the RSPO1-related signature is an independent prognostic factor.

### 3.6. Construction of Nomogram

**Figure 7:**
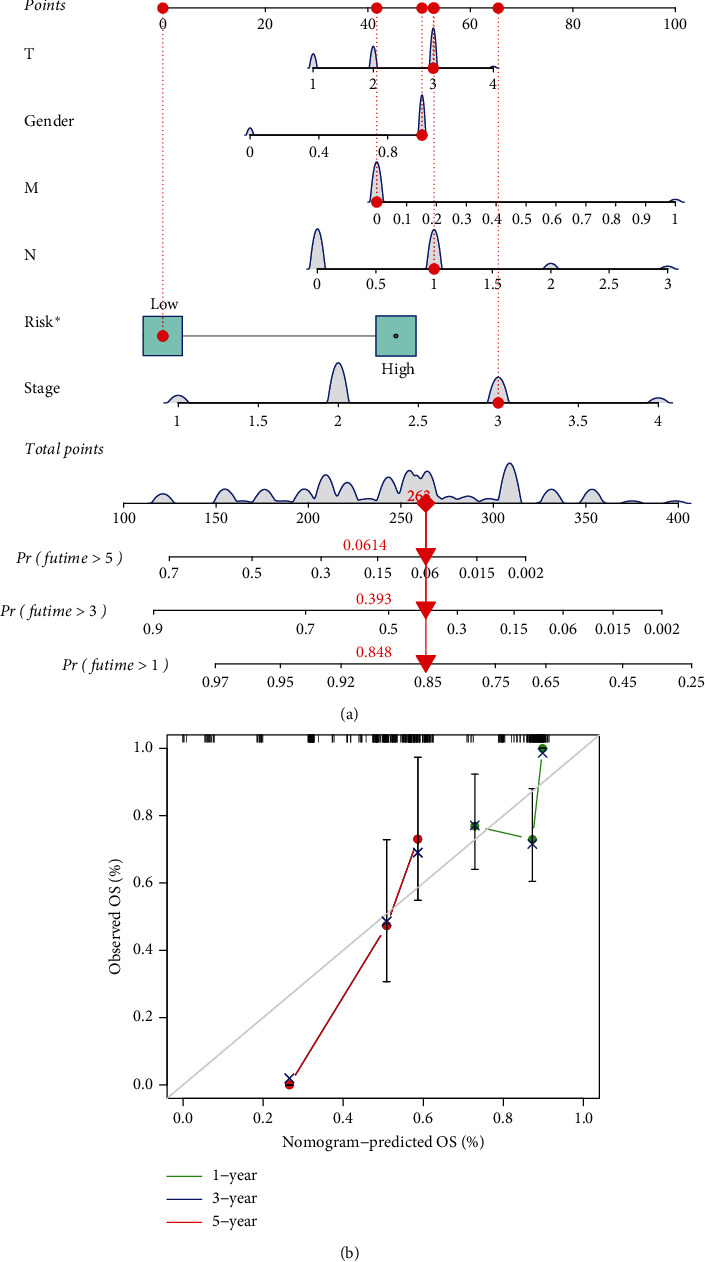
Construction of nomogram in ESCA. (a) A nomogram for predicting 1-, 3- and 5-year survival possibilities of individual ESCA patients. (b) The calibration curve of 1-year, 3-year, and 5-year survival of ESCA.

A nomogram was established to estimate the survival probability of patients with ESCA based on risk, stage, age, T, N, M, and sex (Figure 7(a)). The accuracy was achieved by calibrating the nomogram. The calibration curve revealed that the nomogram accurately predicted the 1-year, 3-year, and 5-year survival rates (Figure 7(b)).

### 3.7. Construction of Gene Coexpression Networks and Enrichment Analysis

**Figure 8:**
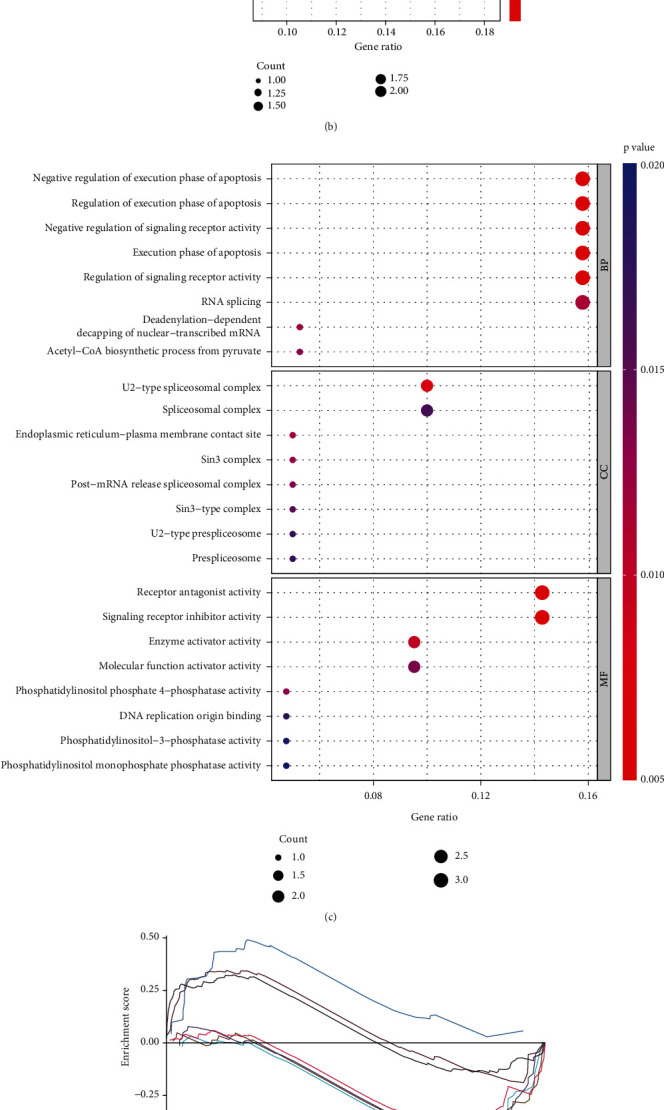
Enrichment analysis and GSEA analysis. (a) The RSPO1-coexpressed genes were visualized using network diagrams. (b) KEGG enrichment analysis was performed on RSPO1-coexpressed genes. (c) GO enrichment analysis of RSPO1-coexpressed genes. (d, e) The GSEA of ESCA cell lines in the CCLE database was used to analyze the signaling pathways of RSPO1-coexpressed genes.

The protein-protein interaction network analysis diagram showed that RSPO1 was coexpressed with the CCLE data. A network of 44 genes coexpressed with RSPO1 is shown in Figure 8(a). We then used the 44 genes coexpressed between RSPO1 and TCGA data for GO and KEGG enrichment analyses. The KEGG results indicated that RSPO1-coexpressed genes were primarily enriched in the cell cycle, spliceosome, Hippo signaling pathway, and citrate cycle (Figure 8(b)). GO results indicated that these coexpressed genes were significantly associated with negative regulation of the execution phase of apoptosis, regulation of the execution phase of apoptosis, negative regulation of signaling receptor activity, regulation of signaling receptor activity, U2-type spliceosomal complex, receptor antagonist activity, signaling receptor inhibitor activity, and so on (Figure 8(c)). GSEA pathway enrichment analysis is essential to elucidate the biological roles of RSPO1. The enrichment results demonstrated a positive correlation between RSPO1-coexpressed genes and kegg_glycolysis_gluconeogenesis, kegg_intestinal_immune_network_for_iga_production, kegg_oxidative_phosphorylation, gobp_cd40_signaling_pathway, gocc_immunoglobulin_complex, and gobp_negative_regulation_of_execution_phase_of_apoptosis and a negative correlation with kegg_p53_signaling_pathway, kegg_renal_cell_carcinoma, kegg_renal_cell_carcinoma, kegg_colorectal_cancer, gobp_hippo_signaling, gomf_ubiquitin_like_protein_specific_protease_activity, and gobp_phosphatidylinositol_3_phosphate_biosynthetic_process (Figures 8(d) and 8(e) and Supplementary Figure 2). GSEA and KEGG showed that RSPO1 was associated with tumor and immune pathways.

### 3.8. ceRNA Network in ESCA

**Figure 9:**
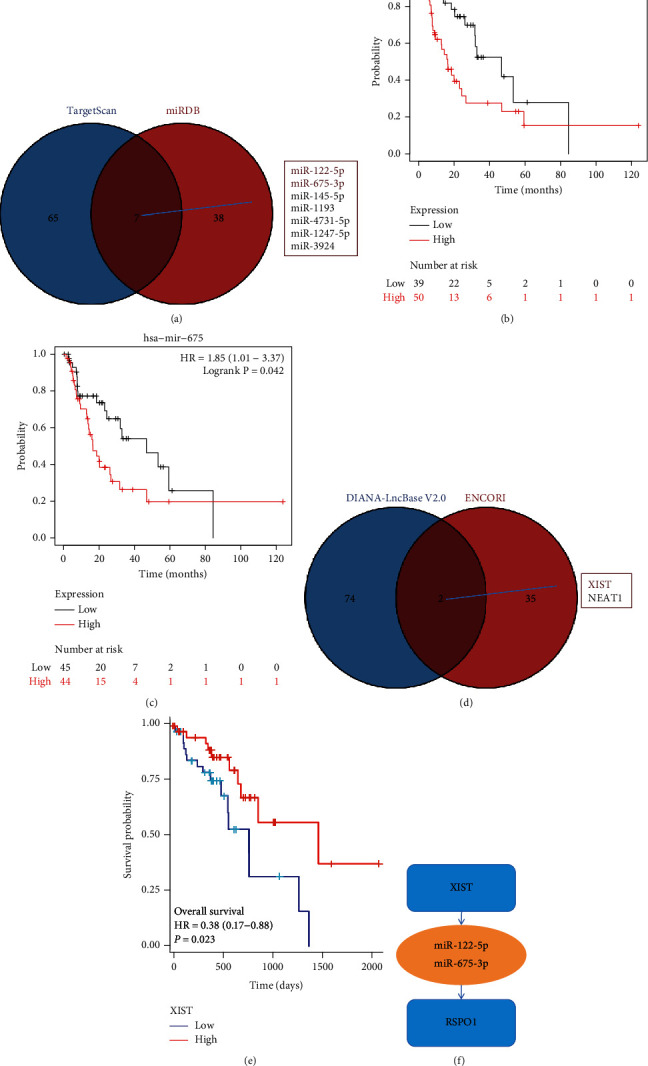
ceRNA network in ESCA. (a) Venn diagram of miRNA intersection. (b, c) Survival analysis of miR-122 and miR-675. (d) Venn diagram of lncRNA intersection. (e) Survival analysis of XIST. (f) ceRNA network of RSPO1 in ESCA.

To explore the relevant mechanisms of RSPO1 ceRNA, we used TargetScan and miRDB to predict target miRNAs of RSPO1. The intersection of the two databases yielded seven miRNAs, among which only miR-122-5p and miR-675-3p showed survival significance in ESCA (Figures 9(a), 9(b), and 9(c)). Subsequently, we used DIANA-LncBase V2.0 and ENCORI to predict the target lncRNAs of miR-122-5p and miR-675-3p. By examining the intersection of results from the two databases, we identified two lncRNAs (Figure 9(d)). We conducted a survival analysis of the identified lncRNAs and found that only XIST was significantly associated with survival (Figure 9(e)). Finally, a possible mechanism diagram of the ceRNA network was constructed (Figure 9(f)). These results suggest that the XIST-miR-122-5p/miR-675-3p-RSPO1 network might regulate the abnormal expression of RSPO1 in ESCA.

## 4. Discussion

The Food and Drug Administration authorized the use of multiple immune checkpoint antibodies for the treatment of ESCA. Nonetheless, tumor clearance by tumor-specific CD8+ T cells was a complex multistep process involving antigen presentation, T cell activation, tumor cell delivery, and tumor cell death [23]. Immunotherapy efficacy may be affected if any of these processes are incorrect. Consequently, the selection of effective immunotherapeutic drugs continues to pose obstacles. Improvement of ESCA therapy could be greatly aided by biomarkers that precisely identify the immunological status of patients.

Our initial analysis focused on individual patient intratumoral immune subsets, as multiple immunotherapies have been developed to control these cells. T lymphocyte subsets, including CD8+ cells, served as potential markers of immunotherapy response [24, 25]. In the ESCA TME, the makeup of 22 immune subsets exhibited significant disparities compared with normal tissues. This technique has enabled the comprehensive exploration of immune-invading cell involvement in ESCA [26, 27], revealing the profound influence of intratumoral infiltration patterns on ESCA prognosis. However, obtaining infiltrating immune cells from individual patients in clinical practice is highly challenging, underscoring the importance of identifying molecular biomarkers to assess a patient's immune status. In this study, GO enrichment analysis of RSPO1-coexpressed genes showed that RSPO1 was closely related to the execution phase of apoptosis, negative regulation of signaling receptor activity, regulation of signaling receptor activity, U2-type spliceosomal complex, receptor antagonist activity, and signaling receptor inhibitor activity. Evidence from KEGG pathway enrichment analysis and GSEA indicated that RSPO1 participates in a number of different biological processes, including the cell cycle, Hippo pathway, intestinal immune network for IgA production, p53 signaling pathway, toll-like receptor signaling pathway, renal cell carcinoma, and colorectal cancer. Interestingly, some studies showed that RSPO1 can suppress colon cancer metastasis by activating TGF*β* signaling [28]. RSPO1 expression in mice inhibited the growth of intestinal adenomas by altering Wnt signaling [29]. Therefore, previous research was consistent with our findings, namely, that RSPO1 and TGF *β* signaling and Wnt signaling pathways are related.

TME features were widely acknowledged as reliable biomarkers for assessing immunotherapy efficacy and influencing prognosis [30]. Additionally, the ESTIMATE algorithm revealed that the high RSPO1 expression group had higher immune, stromal, and estimated ESCA scores than the low RSPO1 expression group. This observation underscores the potential immunotherapeutic effects of high RSPO1 expression, indicating its correlation with improved TME conditions. Therefore, we deduced that reduced RSPO1 expression might be related to immune cell infiltration [31].

Naïve B cells were positively correlated with gene expression, not with copy number variation, but with the downregulation of genes. By contrast, M2 macrophages were negatively correlated with RSPO1 expression, but not with copy number variation, and with gene downregulation. Notably, dendritic cell activation was negatively correlated with RSPO1 expression, copy number variation, and gene downregulation. The proportion of immune cells in the TME showed that the number of tumor-infiltrating immune cells increased significantly. Previous studies have shown that macrophages and dendritic cells are activated and positively correlated with ESCA progression [32–34], whereas resting mast cells are negatively correlated with ESCA progression [35]. Moreover, RSPO1 and RSPO3 expression exhibited significant correlations with immune checkpoint-related genes in various tumors [36], hinting at their potential for the development of immune checkpoint blockade therapy.

Together, the findings suggest that RSPO1 may have important links to tumor immunity.

Immune checkpoints participated in tumor immunosuppression and were considered ideal regulatory targets for tumor immunotherapy [37]. The treatment of immune checkpoints showed strong clinical potential in some patients with cancer but might be hindered by the inability to reverse the immunosuppressive TME. RSPO1/Lgr4 promotes M2 polarization of macrophages by activating the Erk/Stat3 pathway [38]. Our findings confirm the aforementioned results to some extent. Notably, RSPO1 expression was positively correlated with TIGIT, BTLA, CD28, LAIR1, CD40LG, CD27, and CD48 expression in ESCA. RSPO1 expression was strongly correlated with immune checkpoints in ESCA. Based on these findings, we speculate that RSPO1 might predict the state of activated M0 macrophages and dendritic cells and may become a molecular marker for immunotherapy.

The accessibility of publicly available databases could pave the way for more robust and reliable ESCA biomarker identification. Numerous studies have attempted to establish gene expression-based signatures as prognostic indicators of ESCA [39–42]. Consistent with RSPO1-related genes, we established prognostic gene signatures for ESCA. The gene signature-derived risk scores were significantly associated with ESCA survival. The constructed RSPO1-related gene signature contained two prognostic genes (*TECRP1* and *TBK1*) in ESCA. *TECRP1*, a pseudogene, and *TBK1*, a protein-coding gene, are potentially significant factors. TBK1-related diseases include cancer therapy, inflammation, autophagy, acute infectious diseases, and frontotemporal dementia [43, 44]. Currently, there was no research suggesting a direct relation between RSPO1, TECRP1, and TBK1, and the specific regulatory relation requires further study. Finally, we developed a nomogram for prognostic forecasting. Our results indicate that risk assessments based on RSPO1-related genes can differentiate between cancers with high and low risk, potentially contributing to the creation of well-validated signatures for cancer prognosis.

However, some limitations of this study should be noted. First, although we performed some validations, most of the analyses were performed using public datasets. These findings required further validation in an internal patient population. Second, the mechanism of RSPO1-mediated tumor immunity and the prognostic significance of immunological signals required further investigation.

In conclusion, our findings strongly suggest that RSPO1 plays a pivotal role in the tumor immune microenvironment. Prognostic factors derived from RSPO1-linked genes predict ESCA survival, and the clinical application of this biomarker warrants further prospective research.

## Data Availability

The dataset used and analyzed in this study will be provided from the corresponding authors.

## Abbreviations

ESCA: Esophagus cancer
RSPO1: R-spondin 1
OS: Overall survival
CI: Confidence intervals
TCGA: The Cancer Genome Atlas
HR: Hazard ratio
LRP6: Lipoprotein receptor-associated protein 6
TICs: Tumor-infiltrating immune cells
CCLE: Cancer Cell Line Encyclopedia
TME: Tumor microenvironment
GSEA: Gene set enrichment analysis
ROC: Receiver operating characteristic
DCA: Decision curve analysis.

## Supplementary Materials

Supplementary 1 Figure 1: Spearman correlation analysis of RSPO1, TECRP1, and TBK1.

Supplementary 2 Figure 2: the GSEA was used to analyze the GO and KEGG signaling pathways of RSPO1-coexpressed genes in the TCGA database.
