# Supplementary figures and images for: Identification and Validation of Immune Implication of R-Spondin 1 and an R-Spondin 1-Related Prognostic Signature in Esophagus Cancer
Source: Int J Genomics. 2024 May 29;2024:7974277. doi: 10.1155/2024/7974277 (PMC11222003; doi:10.1155/2024/7974277)

Supplementary Figure 1

A

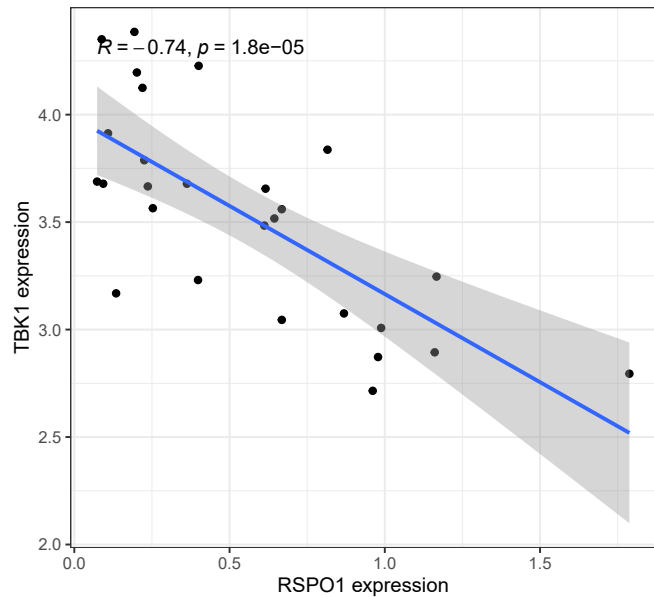

B

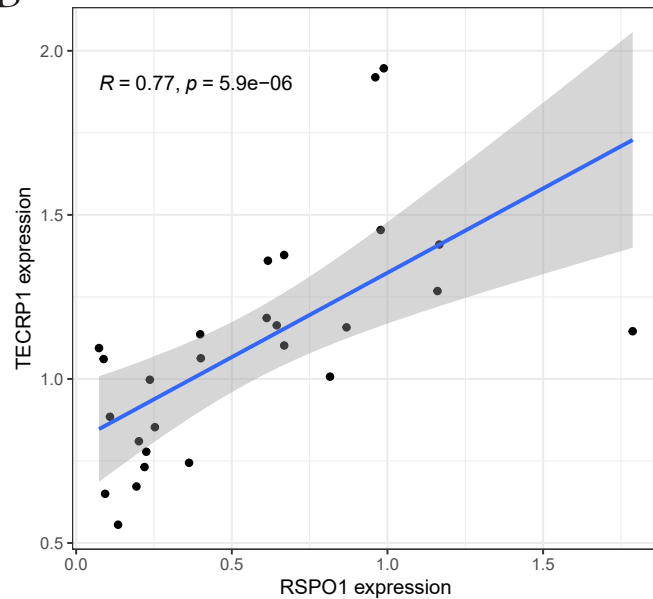

Supplement: Supplementary 1 — Figure 1: Spearman correlation analysis of RSPO1, TECRP1, and TBK1. [file 7974277.f1.pdf]

# Supplementary Figure 2

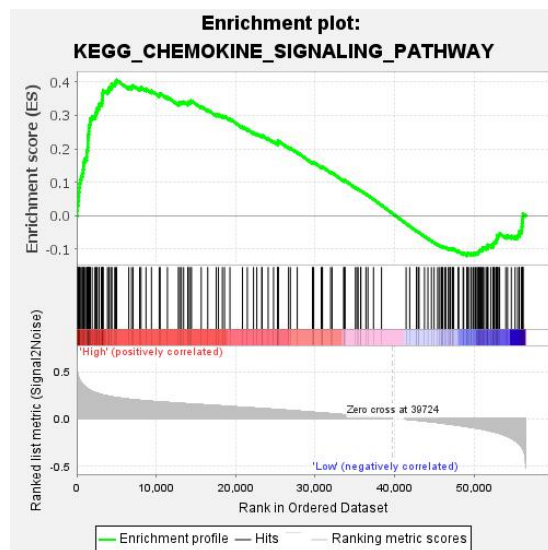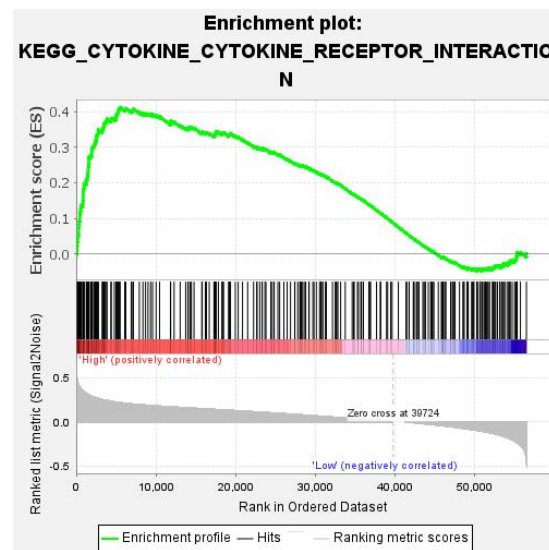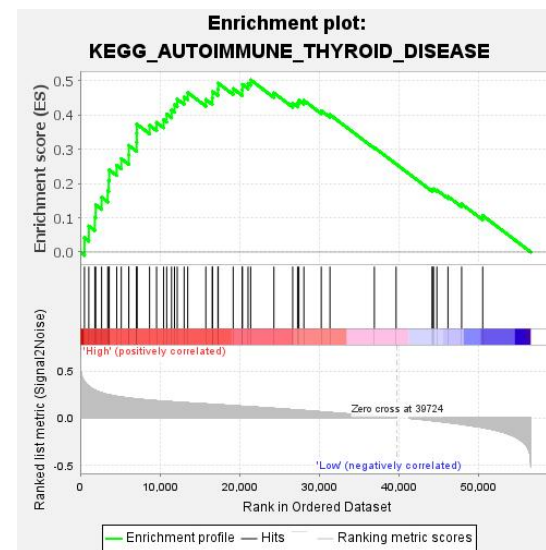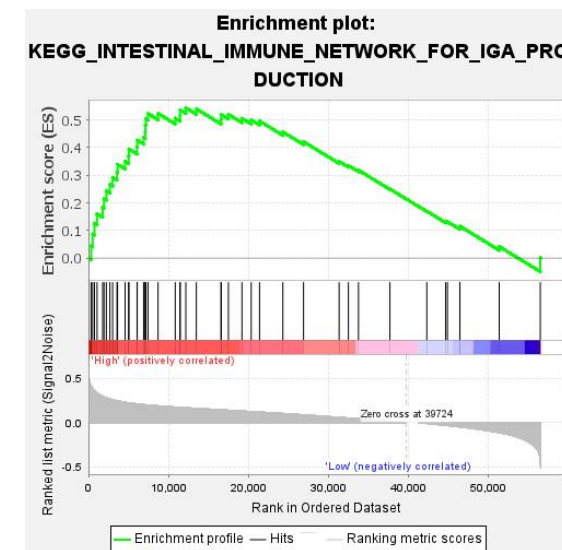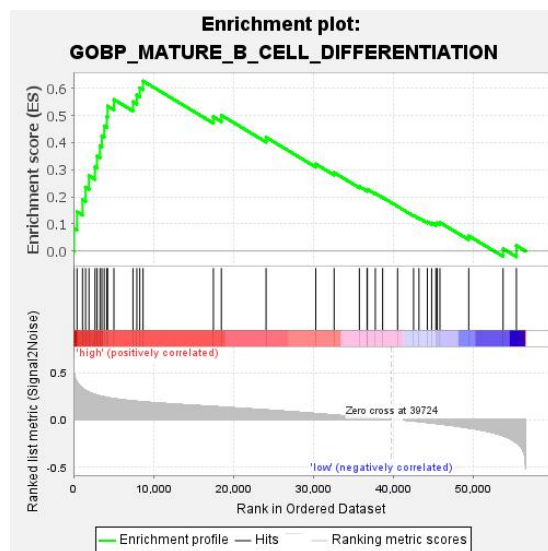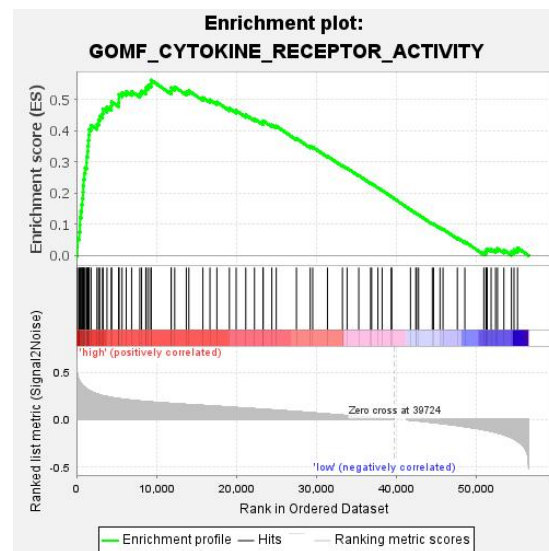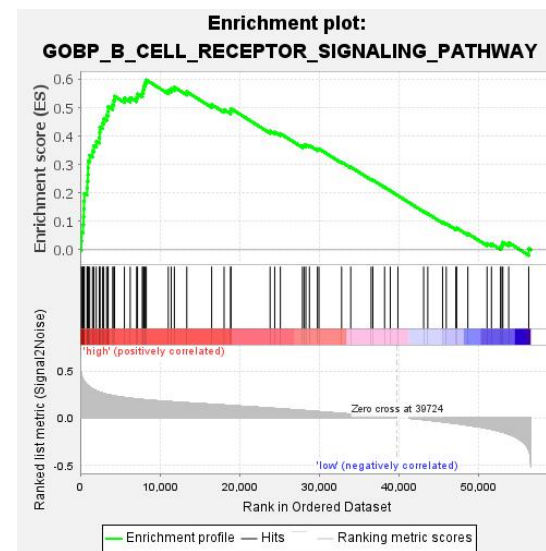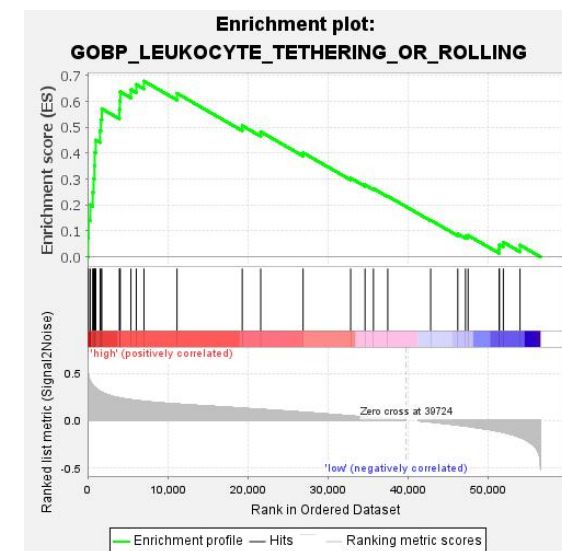

Supplement: Supplementary 2 — Figure 2: the GSEA was used to analyze the GO and KEGG signaling pathways of RSPO1-coexpressed genes in the TCGA database. [file 7974277.f2.pdf]
